# Early diagnosis of acute myocardial infarction using high-sensitivity troponin I

**DOI:** 10.1371/journal.pone.0174288

**Published:** 2017-03-23

**Authors:** Johannes Tobias Neumann, Nils Arne Sörensen, Francisco Ojeda, Thomas Renné, Renate B. Schnabel, Tanja Zeller, Mahir Karakas, Stefan Blankenberg, Dirk Westermann

**Affiliations:** 1 Department of General and Interventional Cardiology, University Heart Center, Hamburg, Germany; 2 German Center for Cardiovascular Research (DZHK), Partner Site Hamburg/Kiel/Lübeck, Germany; 3 Institute of Clinical Chemistry and Laboratory Medicine, University Medical Center Hamburg-Eppendorf, Hamburg, Germany; 4 Clinical Chemistry, Department of Molecular Medicine and Surgery, Karolinska Institute, Stockholm, Sweden; GERMANY

## Abstract

**Objective:**

There is a clinical need for early and accurate diagnosis of acute myocardial infarction (AMI). Current European Society of Cardiology (ESC) guidelines recommend diagnosis of non-ST-elevation AMI based on serial troponin measurements. We aimed to challenge the ESC guidelines using 1) a high-sensitivity troponin I (hs-TnI) baseline cutoff, 2) an absolute hs-TnI change after 1 hour and 3) additional application of an ischemic ECG.

**Methods:**

1,516 patients with suspected AMI presenting to the emergency department were included. Hs-TnI was measured directly at admission, after 1 and 3 hours. We investigated baseline concentrations, absolute changes of hs-TnI and additional application of an ischemic ECG to diagnose AMI. A positive predictive value (PPV) of more than 85% was targeted.

**Results:**

The median age of the study population was 65 years; 291 patients were diagnosed with AMI. The PPV of the 3-hours ESC algorithm was 85.5% (CI 79.7, 90.1) and 65.8% (CI 60.5,70.8) for the 1-hour algorithm. Using a high baseline hs-TnI concentration of 150 ng/L resulted in a PPV of 87.8% (CI 80.9,92.9). Alternatively, a hs-TnI change of 20 ng/L after 1 hour, resulted in a PPV of 86.5% (80.9,91.0), respectively for the diagnosis of AMI. Additional use of an ischemic ECG increased the PPV to 90.5% (CI 83.2,95.3), while reducing the efficacy.

**Conclusion:**

The diagnosis of AMI based on hs-TnI is challenging. The application of absolute hs-TnI changes after 1 hour may facilitate rapid rule-in of patients.

**Trial registration:**

www.clinicaltrials.gov (NCT02355457).

## Introduction

The early diagnosis of acute myocardial infarction (AMI) is highly important in the emergency department (ED).[1,2] Patients with acute onset chest pain frequently present to the ED, while only 15–20% of all patients actually have acute myocardial injury.[3] Earlier studies showed, that an early invasive approach is able to improve outcome in AMI patients.[1,2] On the other hand, invasive therapy itself is associated with higher costs and possible complications.[4–6] This makes the accurate identification of AMI patients crucial.

The measurement of cardiac troponin is gold standard to distinguish between AMI and non-AMI patients.[7] Current ESC guidelines recommend serial measurement of troponin after 1 or 3 hours, when using high-sensitivity assays.[7] The established diagnostic approach is based on the assay-specific 99th percentile in combination with an absolute or relative change of troponin concentration. Earlier studies reported an improved diagnostic performance for absolute changes.[8,9] Application of newer and more sensitive troponin assays enables the detection of much lower concentrations.[10,11] Using a high-sensitivity troponin I (hs-TnI) assay a 3-hours ESC algorithm based on the 99th percentile resulted in a high positive predictive value (PPV) of 83.5%.[12] Application of very low cutoff concentration far below the 99th percentile, a rapid rule-in of AMI after only 1 hour has been investigated.[13–15] In the Biomarkers in Acute Cardiac Care (BACC) study, a 1-hour increase of 12 ng/L was suggested and resulted in a PPV of 87%.[13] In the APACE study a baseline hs-TnI above 52 ng/L or an absolute increase of 6 ng/L from admission to 1 hour have been suggested and resulted in a high PPV of 75%.[15] Importantly, these cutoff concentrations are specific for this hs-TnI assay. Both algorithms enabled the early identification of 55–80% of all AMI patients. The 2015 ESC guideline incorporates a 1-hour approach and enables rapid rule-in of AMI based on low high-sensitivity troponin concentrations.

The aim of our study was to challenge current ESC guideline recommendations and improve the performance to diagnose AMI after only 1 hour using hs-TnI and ECG.

## Methods

### Study population

We used the updated Biomarkers in Acute Cardiac Care (BACC) study population, which has been described previously in detail.[13,16] In summary, 1,641 patients with suspected AMI presenting to the ED or chest pain unit of the University Hospital Hamburg-Eppendorf between 19.07.2013 and 01.04.2016 were included. All individuals were above the age of 18 and gave written informed consent. Patients with ST-elevation myocardial infarction, missing hs-TnI concentrations or missing ECG information were excluded from the analyses (n = 125). The study was approved by the local Ethics Committee (Ethics committee of the Ärztekammer Hamburg, Germany), registered at www.clinicaltrials.gov (NCT02355457) and complied with the Declaration of Helsinki. The assessment and documentation of clinical parameters and cardiovascular risk factors has been described before.[13]

### Clinical standard of care

All patients were diagnosed and treated according to the 2011 ESC guidelines.[17] Blood sampling and ECG analysis were performed at admission and at 3 hours. Troponin T was measured using a high-sensitivity troponin T assay (Elecsys® troponin T high sensitive, Roche Diagnostics). AMI diagnosis was based on troponin T concentrations, clinical symptoms and imaging results and performed by two independent specialists of cardiology. Cases of disagreement were discussed with a third cardiologist. The ECG was written within the ED and acutely interpreted by the emergency physician. A cardiologist reinterpreted the ECG afterwards and based the diagnosis on the universal definition of AMI.[18] The ECG was deemed ischemic, when ST-elevation, ST-deviation, T-wave inversion, ventricular arrhythmias, left or right bundle branch block or > I. grade atrioventricular block was documented.

### High-sensitivity troponin I

Hs-TnI was determined from blood samples collected at admission, after 1 and 3 hours. The sensitivity of the hs-TnI immunoassay (Abbott Diagnostics, USA, ARCHITECT i1000SR) had a limit of detection at 1.9 ng/L (range 0–50,000 ng/L) and a 10 percent coefficient of variation at a concentration of 5.2 ng/L. The assay-specific 99^th^ percentile was described at 27 ng/L in the general population.[19]

### Diagnostic approach to rule-in AMI using high-sensitivity troponin I

We first applied the current recommendations of the ESC and the ESC Working Group on Acute Cardiac Care to our study population.[7,20] This standard 3-hours algorithm was based on the 99th percentile (27 ng/L) and used a relative delta of ≥20% (admission to 3 hours), if the concentration was above the 99th percentile at baseline and after 3 hours, or an absolute delta of ≥50% of the 99th percentile (0.5 × 27 = 13.5 ng/L), if the concentration was at or below the 99th percentile at baseline and above the 99th percentile after 3 hours. The standard 1-hours algorithm included a baseline hs-TnI cutoff concentration of 52 ng/L or an absolute change from 0 to 1 hour of more than 6 ng/L.

In a second step, we aimed to increase the PPV to more than 85%, while still including a maximum of possible AMI patients. For this approach, we calculated the PPV, sensitivity, specificity false and true positive patients (FP, TP) for different algorithms: 1) Different baseline hs-TnI concentrations were investigated. Here, cutoff concentrations of 3, 6, 9, 12, 15, 20, 27, 52, 100, 150, 200, 1.000 and 2.000 ng/L were based on prior publications and aimed to be integer numbers.[13,15] 2) The baseline hs-TnI concentration with a PPV of more than 85% was used in combination with different absolute hs-TnI changes from admission to 1 hour. 3) Again, the baseline hs-TnI concentration with a PPV of more than 85% was used and combined with a 0/1h hour change and the information of an ischemic ECG.

### Statistics

Continuous variables are described by its quartiles, categorical variables, by its absolute and relative frequencies. The different algorithms were based on the hs-TnI measurements (at admission or 1 hour or 3 hours later) and their differences, or deltas, from admission to either 1 hour or 3 hours later. Absolute and relative differences were considered. The absolute difference from admission until 1 hour later is defined as |hs-TnI 1h –hs-TnI 0h| and the relative difference with respect to the admission value is defined as 100 × |hs-TnI 1h –hs-TnI 0h|/(hs-TnI 0h), where || denotes the absolute value function. 3-hour deltas are defined in a similar fashion (exchanging hs-TnI 1h by hs-TnI 3h). For the diagnostic tests considered sensitivity, specificity and PPV were computed. For all these quantities 95% exact binomial confidence intervals were calculated. All analyses were performed using R version 3.3.0 (R Core Team (2016). R: A language and environment for statistical computing. R Foundation for Statistical Computing, Vienna, Austria. URL http://www.R-project.org/).

## Results

### Baseline characteristics

The baseline characteristics of the study population have been described before.[21] Briefly, we included 1,516 patients with suspected AMI. (**Table 1**) 291 patients were diagnosed with AMI. The median age of all patients was 65 (25^th^ and 75^th^ percentile 51.0,75.0) years and 63.7% were male. Cardiovascular risk factors were more common in AMI patients compared to the overall population.

**Table 1:** Baseline characteristics.

	All (N = 1,516)	AMI (N = 291)
Age (years)	65.0 (51.0, 75.0)	70.0 (60.0, 76.0)
Male (%)	966 (63.7)	189 (64.9)
BMI (kg/m^2^)	26.1 (23.6, 29.4)	26.6 (23.7, 29.8)
Hypertension (%)	1,029 (68.2)	230 (79.3)
Hyperlipoproteinemia (%)	597 (39.4)	150 (51.5)
Diabetes (%)	198 (13.2)	53 (18.3)
Former smoker (%)	456 (30.1)	90 (30.9)
Current smoker (%)	347 (22.9)	71 (24.4)
History of CAD/Bypass/PCI (%)	520 (34.3)	125 (43.0)
Atrial fibrillation (%)	264 (17.4)	53 (18.2)
Congestive heart failure (%)	218 (14.4)	59 (20.3)
Creatinine (mg/dL)	1.0 (0.8, 1.2)	1.1 (0.9, 1.3)

AMI = acute myocardial infarction; BMI = body-mass-index; CAD = coronary artery disease; PCI = percutaneous coronary intervention.

### Standard approach

**Fig 1:**
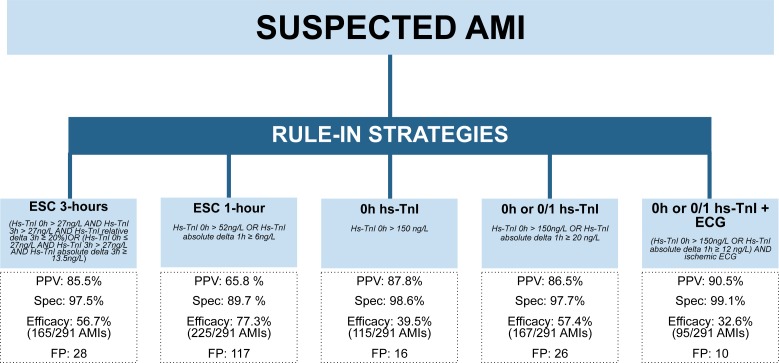
Diagnostic performance of different approaches to rule-in AMI.

Application of the standard 3-hours diagnostic algorithm (27 ng/L cutoff concentration and a relative 20% or 50% change 0/3 hours) resulted in a PPV of 85.5% (Confidence Interval [CI] 79.7, 90.1) to diagnose AMI. (**Fig 1, S1 Table**) This approach included 165 TP (efficacy 56.7%) and only 28 FP patients. Using the 1-hour ESC standard diagnostic approach (0h > 52 ng/L or 6 ng/L change) resulted in a PPV of 65.8% (CI 60.5,70.8) including 225 TP (efficacy 77.3%) and 117 FP patients.

### Baseline measurement to diagnose AMI

Different baseline hs-TnI concentrations were used to calculate the PPV to diagnose AMI. (**Table 2**) Application of a low baseline concentration of 3 ng/L resulted in a PPV of 25.5% (CI 23.0,28.2). Using a cutoff concentration at 27 ng/L (99th percentile) or at 52 ng/L (recommended in ESC guideline) translated to a PPV of 65.4% (CI 59.5,70.9) and 73.8% (CI 67.2,79.7), respectively. A PPV of 87.8% (CI 80.9,92.9), which is above the targeted 85% PPV, was observed at a cutoff concentration of 150 ng/L. This included 115 TP (efficacy 39.5%) and 16 FP patients.

**Table 2:** Rule-in using a single baseline troponin measurement.

Cutoff	Sensitivity	Specificity	PPV	TP+FP	All N
> 3 ng/L	99.6 (98.0, 100.0)	31.2 (28.6, 33.9)	25.5 (23.0, 28.2)	279+814 = 1,093	1,463
> 6 ng/L	92.9 (89.2, 95.6)	56.2 (53.3, 59.1)	33.4 (30.1, 36.9)	260+518 = 778	1,463
> 9 ng/L	87.1 (82.6, 90.8)	72.0 (69.4, 74.6)	42.4 (38.4, 46.6)	244+331 = 575	1,463
> 12 ng/L	81.4 (76.4, 85.8)	79.3 (76.9, 81.6)	48.2 (43.6, 52.8)	228+245 = 473	1,463
> 15 ng/L	78.2 (72.9, 82.9)	84.7 (82.5, 86.7)	54.8 (49.7, 59.7)	219+181 = 400	1,463
> 20 ng/L	72.1 (66.5, 77.3)	88.8 (86.9, 90.6)	60.5 (55.0, 65.8)	202+132 = 334	1,463
> 27 ng/L	66.1 (60.2, 71.6)	91.7 (90.0, 93.2)	65.4 (59.5, 70.9)	185+98 = 283	1,463
> 52 ng/L	54.3 (48.3, 60.2)	95.4 (94.1, 96.6)	73.8 (67.2, 79.7)	152+54 = 206	1,463
> 100 ng/L	46.1 (40.1, 52.1)	97.5 (96.5, 98.4)	81.6 (74.7, 87.3)	129+29 = 158	1,463
> 150 ng/L	41.1 (35.3, 47.1)	98.6 (97.8, 99.2)	87.8 (80.9, 92.9)	115+16 = 131	1,463
> 200 ng/L	38.9 (33.2, 44.9)	99.0 (98.2, 99.5)	90.1 (83.3, 94.8)	109+12 = 121	1,463
> 400 ng/L	32.1 (26.7, 38.0)	99.8 (99.4, 100.0)	97.8 (92.4, 99.7)	90+2 = 92	1,463
> 1,000 ng/L	20.7 (16.1, 25.9)	99.9 (99.5, 100.0)	98.3 (90.9, 100.0)	58+1 = 59	1,463
> 2,000 ng/L	13.6 (9.8, 18.1)	100.0 (99.7, 100.0)	100.0 (90.7, 100.0)	38+0 = 38	1,463

PPV = positive predictive value; TP = true positives; FP = false positives. The numbers in brackets represent 95% confidence intervals.

### Absolute hs-TnI changes after 1 hour

In order to still include a baseline approach, the absolute hs-TnI changes were combined with a baseline hs-TnI concentration of 150 ng/L using an “or” criterion. An absolute change of hs-TnI > 6 ng/L from admission to 1 hour resulted in a PPV of 67.7% (CI 62.3,72.8) for AMI. **(Table 3**) With increasing absolute changes the PPV showed a rapid increase, while the increase was less prominent for higher absolute changes. (**S1 Fig**) The targeted PPV of more than 85% was reached at a 20 ng/L absolute change. This translated to a PPV of 86.5% (CI 80.9,91.0) including 167 TP (efficacy 57.4%) and 26 FP patients.

**Table 3:** Rule-in using a single baseline troponin measurement or a 0/1-hour absolute change.

Hs-TnI 0h ≥ 150 ng/L OR 0/1h delta	Sensitivity	Specificity	PPV	TP+FP	All N
≥ 6 ng/L	80.3 (75.0, 84.9)	91.0 (89.1, 92.6)	67.7 (62.3, 72.8)	216+103 = 319	1,408
≥ 9 ng/L	73.2 (67.5, 78.4)	95.3 (94.0, 96.5)	78.8 (73.2, 83.7)	197+53 = 250	1,408
≥ 12 ng/L	68.4 (62.5, 73.9)	96.8 (95.6, 97.7)	83.3 (77.7, 87.9)	184+37 = 221	1,408
≥ 15 ng/L	65.1 (59.0, 70.7)	96.9 (95.8, 97.9)	83.3 (77.6, 88.1)	175+35 = 210	1,408
≥ 20 ng/L	62.1 (56.0, 67.9)	97.7 (96.7, 98.5)	86.5 (80.9, 91.0)	167+26 = 193	1,408
≥ 27 ng/L	60.6 (54.5, 66.5)	98.2 (97.2, 98.9)	88.6 (83.1, 92.8)	163+21 = 184	1,408
≥ 52 ng/L	52.4 (46.3, 58.5)	98.4 (97.5, 99.1)	88.7 (82.7, 93.2)	141+18 = 159	1,408
≥ 100 ng/L	48.7 (42.6, 54.8)	98.5 (97.6, 99.1)	88.5 (82.2, 93.2)	131+17 = 148	1,408
≥ 150 ng/L	45.7 (39.7, 51.9)	98.6 (97.7, 99.2)	88.5 (82.0, 93.3)	123+16 = 139	1,408

h = hour; PPV = positive predictive value; TP = true positives; FP = false positives. The numbers in brackets represent 95% confidence intervals.

### Ischemic ECG

After addition of the information of an ischemic ECG the PPVs were higher, while the efficacy was reduced. An absolute change of hs-TnI > 6 ng/L from admission to 1 hour resulted in a PPV of 77.0% (CI 69.1,83.7) for AMI. **(Table 4**) Again, increasing absolute changes the PPV showed a rapid increase, while the increase was less strong for absolute changes above 10 ng/L. (**S2 Fig**) A PPV of more than 85% was reached at a 12 ng/L change (PPV 90.5%) and included 95 TP (efficacy 32.6%) and 10 FP patients.

**Table 4:** Rule-in with additional use of an ischemic ECG.

(Hs-TnI 0h ≥ 150 ng/L OR 0/1h delta) AND ischemic ECG	Sensitivity	Specificity	PPV	TP+FP	All N
≥ 6 ng/L	39.8 (33.9, 45.9)	97.2 (96.1, 98.1)	77.0 (69.1, 83.7)	107+32 = 139	1,408
≥ 9 ng/L	37.5 (31.7, 43.6)	98.4 (97.5, 99.1)	84.9 (77.2, 90.8)	101+18 = 119	1,408
≥ 12 ng/L	35.3 (29.6, 41.4)	99.1 (98.4, 99.6)	90.5 (83.2, 95.3)	95+10 = 105	1,408
≥ 15 ng/L	33.1 (27.5, 39.1)	99.2 (98.5, 99.6)	90.8 (83.3, 95.7)	89+9 = 98	1,408
≥ 20 ng/L	31.6 (26.1, 37.5)	99.5 (98.9, 99.8)	93.4 (86.2, 97.5)	85+6 = 91	1,408
≥ 27 ng/L	30.5 (25.0, 36.4)	99.6 (99.1, 99.9)	95.3 (88.5, 98.7)	82+4 = 86	1,408
≥ 52 ng/L	27.1 (21.9, 32.9)	99.6 (99.1, 99.9)	94.8 (87.2, 98.6)	73+4 = 77	1,408
≥ 100 ng/L	25.3 (20.2, 30.9)	99.7 (99.2, 99.9)	95.8 (88.1, 99.1)	68+3 = 71	1,408
≥ 150 ng/L	23.4 (18.5, 28.9)	99.7 (99.2, 99.9)	95.5 (87.3, 99.1)	63+3 = 66	1,408

h = hour; PPV = positive predictive value; TP = true positives; FP = false positives. The numbers in brackets represent 95% confidence intervals.

## Discussion

This study challenges the current 2015 ESC guideline recommendations on the rule-in of AMI patients and aims for a positive predictive value of more than 85%. Different early rule-in strategies were investigated. A high baseline concentration or an absolute change of high-sensitivity troponin I after only 1 hour resulted in a high positive predictive value.

### Standard approach based on 99th percentile

In 2012 the ESC Working Group on Acute Cardiac Care published a recommendation on the use of high-sensitivity troponin to diagnose or to rule-out AMI. [20] A 3- or 6-hours approach was recommended, which is based on the 99th percentile and uses a 20 or 50% change as a cutoff concentration after serial sampling. This approach has recently been validated by Pickering et al, who described a good diagnostic performance to rule-in AMI. [12] Using the same hs-TnI assay as we do, they reported a high PPV of 83.5% in a pooled study population of 1,172 chest pain patients. The sensitivity was 65.2% and only 17 patients were classified being false positive. In the BACC cohort we as well found a high PPV of 85.5% using the same diagnostic approach after 3 hours. The sensitivity was similar (62.0%) and only 28 patients were false positives. This result validates the diagnostic performance in cohorts with different characteristics and pretest probabilities. As an example, Pickering et al reported an AMI rate of 12.4%, while it was 19.0% in the BACC study. These results verify the guidelines recommendations with good PPVs, but this is still associated with a long delay of 3 or 6 hours in respect to the used protocol before the final diagnosis can be made. Coherently, we tested whether a rapid 1-hour rule-in is feasible and as efficient as a longer protocol.

### Rapid approach

An early or immediate invasive approach is also recommended by the ESC guideline in high-risk patients with AMI.[7] The recently published RIDDLE-NSTEMI study showed an improved outcome for those non-ST-elevation AMI patients that underwent coronary intervention within 2 hours after admission.[2] These results underline the importance of early decision-making in patients with suspected AMI and support the development of rapid rule-in protocols. The first 1-hour approach using hs-TnI sampling has been investigated in the APACE cohort, which is now incorporated in the 2015 ESC guideline.[15] Here, an absolute hs-TnI change of 6 ng/L after 1 hour was suggested and resulted in a PPV of 75.6%. The corresponding PPV in the BACC study was only 65.8% including 117 false positive patients. Pickering et al recently reported similar findings in a multicenter setting.[14] Therefore, we suppose this might not be the ideal cutoff for daily clinical use.

We investigated different higher baseline cutoff and absolute delta changes of hs-TnI in order to improve the diagnostic performance of a possible rapid rule-in algorithm. As expected, the number of false positive patients showed a rapid decline with increasing delta. As an example, increasing the absolute change after 1 hour from 6 ng/L to 9 ng/L reduced the false positive patients from 103 to 53, while the sensitivity was still 73.2% and the PPV 78.8%. Higher deltas of 12 ng/L and 27 ng/L increased the PPV to 83.3 and 88.6%. These cutoffs resulted in PPVs, which seem to be as efficient as the 3-hour algorithm suggested by the guidelines, while the sensitivity is still comparable. Therefore, these higher deltas, compared to guideline suggestions, might offer safe and efficient rule-in after 1 hour.

Even a single baseline measurement with a hs-TnI cutoff at 52 ng/L is suggested in the ESC guideline. This cutoff concentration resulted in a PPV of 73.8% and a sensitivity of 54.3% in our cohort including 54 false positive patients. Again, higher cutoff concentrations increased the PPV, while the sensitivity was reduced.

Taken together, these findings raise the question, of how sure we want to be regarding the rule-in of patients. Should we aim for rule-in algorithm with a high PPV in order not to potentially harm patients due to invasive management, or should we aim for a high sensitivity in order not to miss any patients?

Pickering et al discussed a PPV of around 80%, which would be accepted by most clinicians, as it includes only 20% false positive patients. In our present study, we aimed for a PPV of more than 85%, as this was achieved by the clinical standard using the 3-hours approach. This approach is highly accepted by clinicians and used worldwide. However, most investigations are solely based on biomarker analyses and dismiss the actual clinical symptoms, cardiovascular risk profile and other diagnostic tools, such as ECG or echocardiography. To address this topic, we included the information of an ischemic ECG to the analyses. Here, the PPVs were increased, while the efficacy was reduced. As an example, the PPV was increased from 83.3% to 90.5%, when adding the ischemic ECG to an absolute change of 12 ng/L. However, the TP patients were reduced from 184 to 95. These findings highlight the importance of troponin concentrations, when diagnosing AMI. Nevertheless, physicians in the ED need to add further information to their decision-making, when using high-sensitivity troponin assays. In future, an even more individualized approach that is not based on clear cutoff concentrations, but includes a variety of risk predictors might be useful to optimize the diagnostic accuracy.

### Strengths and limitations

This study has several strengths, but also some limitations. The BACC study is a well-characterized population with a gold-standard adjudication of the final diagnosis and serial sampling after only 1 hour. Nevertheless, it is a single-center study and represents a limited variety of patients. The results presented in this manuscript are specific for this hs-TnI assay and cannot be generalized to other assays. Further validation in external cohorts and other troponin assays is therefore mandatory. Finally, our analyses were performed in a prospective, but not randomized study. Therefore, it remains a scientific need to compare current clinical standard to a rapid approach in a randomized trial.

## Conclusion

The diagnosis of AMI based on hs-TnI is still challenging. Final decision-making should always include individual the cardiovascular risk profile and other diagnostic tools. The application of absolute hs-TnI changes after 1 hour might facilitate rapid rule-in of AMI patients.

## Supporting information

S1 Fig Positive predictive value for diagnosis of AMI using a single baseline troponin measurement or a 0/1-hour absolute change. A patient is declared to have AMI if Hs-TnI 0h > 150ng/L or Hs-TnI absolute delta 1h ≥ cut-off. Only cut-offs ≤ 60ng/L are used to produce the graphic.
(PNG)

S2 Fig Positive predictive value for diagnosis of AMI after additional use of ischemic ECG. A patient is declared to have AMI if (Hs-TnI 0h > 150ng/L or Hs-TnI absolute delta 1h ≥ cut-off) and ischemic ECG. Only cut-offs ≤ 60ng/L are used to produce the graphic.
(PNG)

S1 Table Diagnostic performance following the standard 1- and 3-hours ESC-algorithm to diagnose AMI (DOC)
