# Supplementary figures and images for: Early diagnosis of acute myocardial infarction using high-sensitivity troponin I
Source: PLoS One. 2017 Mar 23;12(3):e0174288. doi: 10.1371/journal.pone.0174288 (PMC5363912; doi:10.1371/journal.pone.0174288)

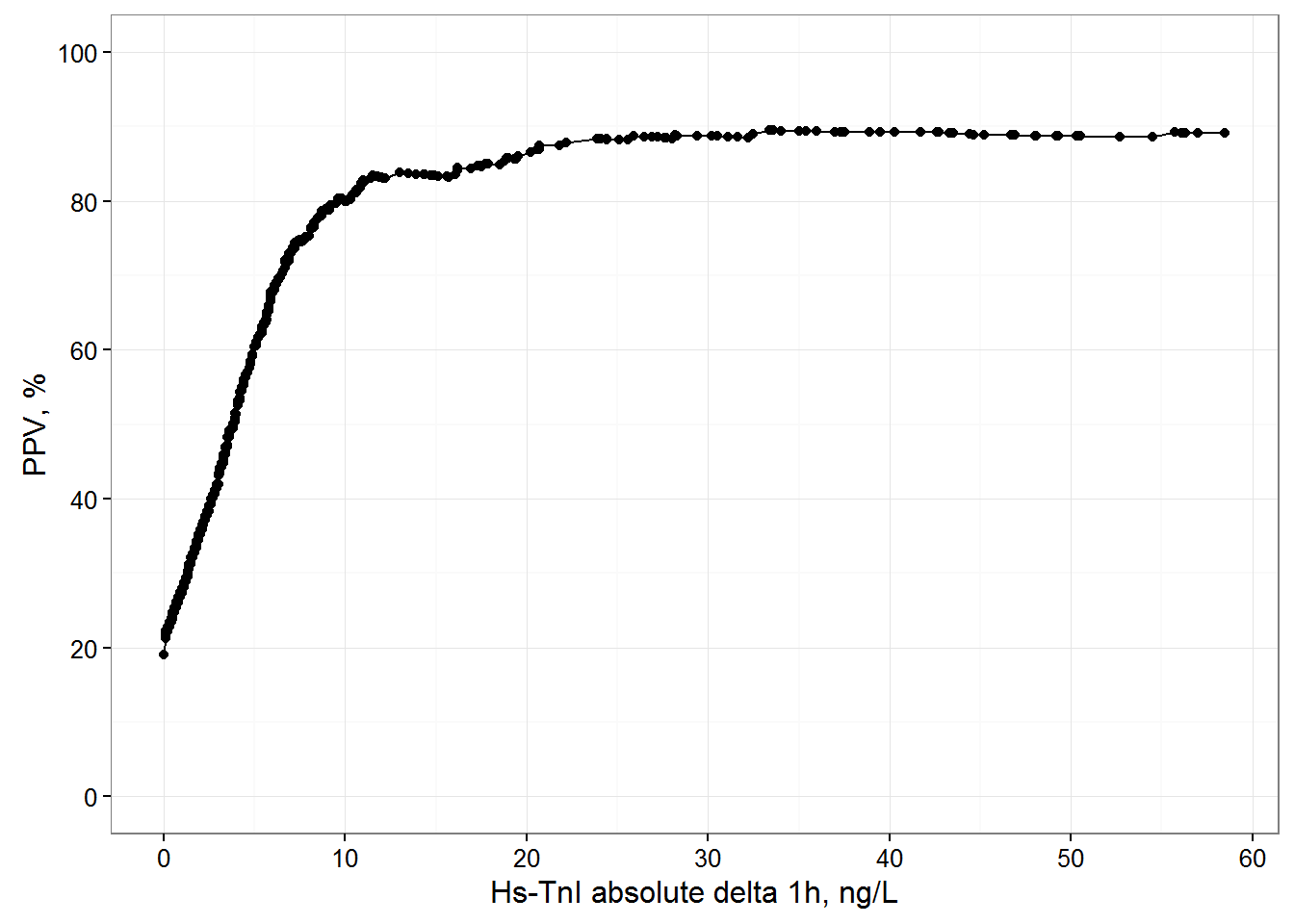

Supplement: S1 Fig — A patient is declared to have AMI if Hs-TnI 0h > 150ng/L or Hs-TnI absolute delta 1h ≥ cut-off. Only cut-offs ≤ 60ng/L are used to produce the graphic. (PNG) [file pone.0174288.s001.png]

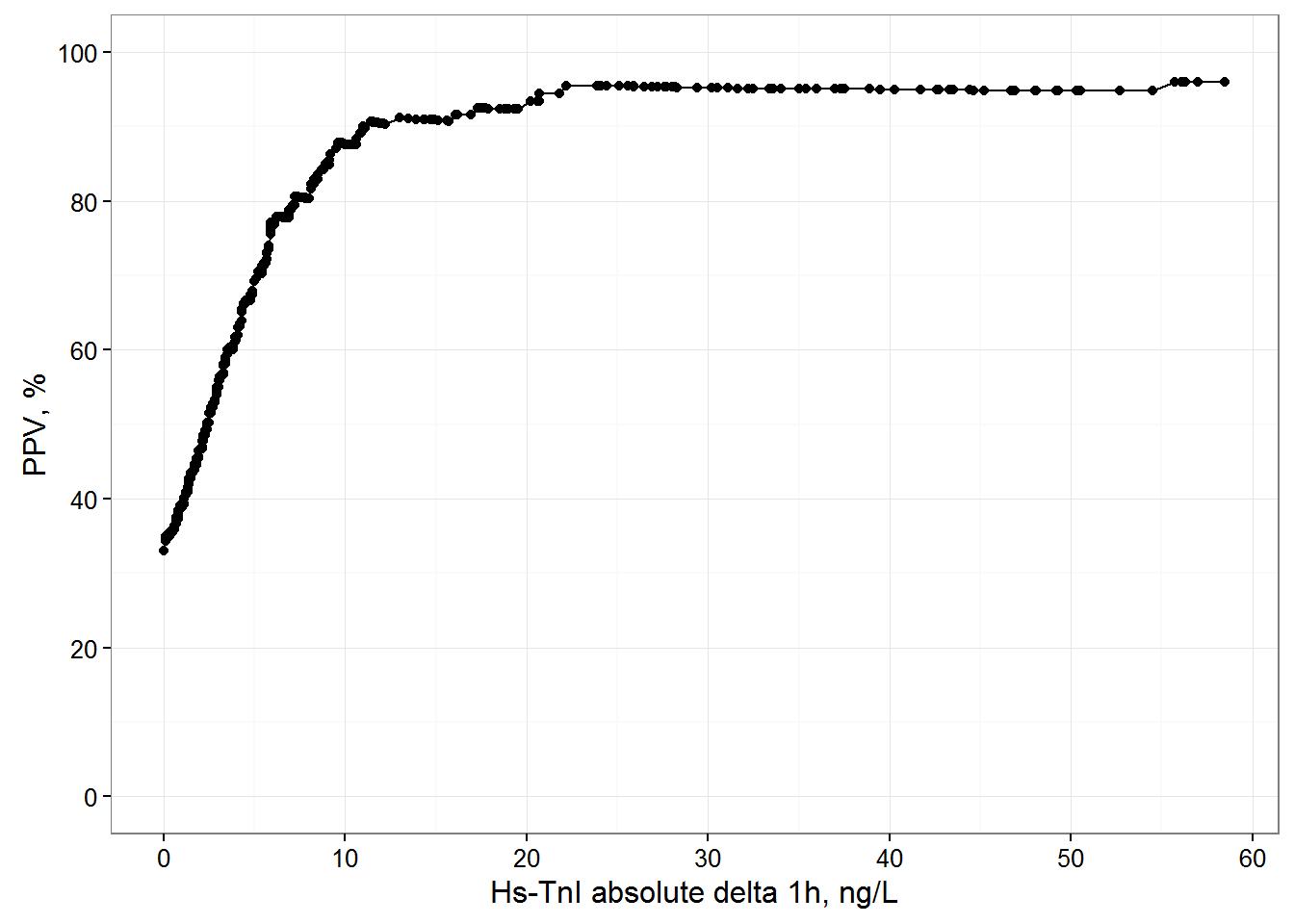

Supplement: S2 Fig — A patient is declared to have AMI if (Hs-TnI 0h > 150ng/L or Hs-TnI absolute delta 1h ≥ cut-off) and ischemic ECG. Only cut-offs ≤ 60ng/L are used to produce the graphic. (PNG) [file pone.0174288.s002.png]
