# Supplementary material for: Early diagnosis of acute myocardial infarction using high-sensitivity troponin I
Source: PLoS One. 2017 Mar 23;12(3):e0174288. doi: 10.1371/journal.pone.0174288 (PMC5363912; doi:10.1371/journal.pone.0174288)
Supplement: S1 Table — (DOC) [file pone.0174288.s003.doc]

**S1 Table: Diagnostic performance following the standard 1- and 3-hours ESC-algorithm to diagnose AMI**

|  | **Sensitivity** | **Specificity** | **PPV** | **TP+FP** | **All N** |
| --- | --- | --- | --- | --- | --- |
| **1-hour** | 83.6 (78.7, 87.9) | 89.7 (87.8, 91.4) | 65.8 (60.5, 70.8) | 225+117=342 | 1,408 |
| **3-hours** | 62.0 (55.9, 67.9) | 97.5 (96.5, 98.4) | 85.5 (79.7, 90.1) | 165+28=193 | 1,403 |

AMI = acute myocardial infarction; PPV= positive predictive value; TP = true positives; FP = false positives. The numbers in brackets represent 95% confidence intervals.
